# Dorsal and Ventral Hippocampus Differentiate in Functional Pathways and Differentially Associate with Neurological Disease-Related Genes during Postnatal Development

**DOI:** 10.3389/fnmol.2017.00331

**Published:** 2017-10-16

**Authors:** A-Ram Lee, Jong-Hwan Kim, Eunsil Cho, Mirang Kim, Mikyoung Park

**Affiliations:** ^1^Center for Functional Connectomics, Brain Science Institute, Korea Institute of Science and Technology, Seoul, South Korea; ^2^Personalized Genomic Medicine Research Center, Korea Research Institute of Bioscience and Biotechnology, Daejeon, South Korea; ^3^Department of Functional Genomics, Korea University of Science and Technology, Daejeon, South Korea; ^4^Department of Neuroscience, Korea University of Science and Technology, Daejeon, South Korea

**Keywords:** dorsal hippocampus, ventral hippocampus, transcriptome, RNA-seq, neurological diseases, postnatal development

## Abstract

The dorsal and ventral regions of the hippocampus are important in cognitive and emotional processing, respectively. Various approaches have revealed the differential molecular and structural characteristics, and functional roles of the hippocampus. Recent RNA sequencing (RNA-seq) technology has enriched our understanding of the hippocampus by elucidating more detailed information on gene expression patterns. However, no RNA-seq–based study on gene profiles in the developing hippocampus has been reported. Using RNA-seq–based bioinformatic analysis in conjunction with quantitative real-time polymerase chain reaction analysis and a comparison of *in situ* hybridization data obtained from the Allen Brain Atlas, we provide a thorough analysis of differentially expressed genes in the dorsal and ventral hippocampus at specific developmental ages representing the postnatally maturing hippocampus. Genes associated with particular functional pathways and marker genes for particular neurological diseases were found to be distinctively segregated within either the dorsal or ventral hippocampus at specific or at all developmental ages examined. We also report novel molecular markers enriched in the dorsal or ventral hippocampus. Taken together, this study provides insights into the molecular mechanisms underlying physiological functions linked to the dorsal or ventral hippocampus. The information provided in the study also contributes to a better understanding of brain functions and serves as a resource for future studies on the pathophysiology of dorsal and ventral hippocampal functions.

## Introduction

The hippocampus has traditionally been explored for its function in memory formation (Eichenbaum, 1997; Squire et al., 2004). However, this region has also been proposed to regulate other functions in the brain (Fanselow and Dong, 2010). Although the basic circuitry of the hippocampus is remarkably similar along its septotemporal axis, the main intrinsic and extrinsic connections are very different for the dorsal and ventral regions, and they connect with different sets of extra-hippocampal structures (Ruth et al., 1982; Roberts et al., 1984; Van Groen and Lopes da Silva, 1985; Witter, 1986; Bannerman et al., 2014). Whereas the dorsal hippocampus receives polymodal sensory information from cortical areas, the ventral hippocampus is much more closely linked to subcortical structures, such as the amygdala, and the hypothalamic-pituitary-adrenal axis (Bannerman et al., 2014).

Research on the region-specific functional differentiation within the hippocampus has recently extended from experimental analyses to large-scale bioinformatics analyses (Moser and Moser, 1998; Leonardo et al., 2006; Lein et al., 2007; Thompson et al., 2008; Dong et al., 2009; McHugh et al., 2011). The dorsal hippocampus has been reported to be responsible for cognitive processing, such as spatial learning (Kim and Fanselow, 1992; Moser et al., 1993, 1995; McHugh et al., 2011; Bannerman et al., 2014), while the ventral hippocampus has been suggested to be involved in emotional processing, such as anxiety (Bannerman et al., 2003, 2004, 2014; McHugh et al., 2011). Different subtypes of neurons in the dorsal and ventral hippocampus were distinctly responsive to chronic stress (Czéh et al., 2015). In addition, chronic stress led to a decrease in long-term depression in the ventral but not dorsal hippocampus, further revealing a region-specific impact of chronic stress (Pinto et al., 2015). Furthermore, stress and corticosteroids differentially modulate long-term potentiation and long-term depression along the septotemporal axis of the hippocampus (Maggio and Segal, 2007a,b, 2009a,b, 2010, 2011). Compared with the dorsal hippocampus, the ventral hippocampus expresses lower magnitude long-term potentiation. Exposure to acute stress reversed this difference, and the ventral hippocampus from stressed rats expressed greater long-term potentiation than that produced in the dorsal hippocampus, which showed stress-induced reduction in long-term potentiation (Maggio and Segal, 2007b, 2009b, 2010, 2011). Differential sensitivity in responses to stimuli inducing NMDA-mediated potentiation has also been reported in the dorsal and ventral hippocampus (Maggio et al., 2015).

Such functional and behavioral differentiation within the hippocampus implies region- and cell-specific molecular heterogeneity. Indeed, serotonin receptors were shown to be differentially expressed in different regions of the hippocampus (Tanaka et al., 2012). Microarray analyses have also revealed the molecular heterogeneity of the hippocampus along the dorsal-ventral axis (Leonardo et al., 2006; O’Reilly et al., 2015). More recent work has recapitulated and extended the previous research on the differentiation of the hippocampus along the dorsal-ventral axis (Zeisel et al., 2015; Cembrowski et al., 2016a,b) by using RNA sequencing (RNA-seq), a powerful tool that enables systematic, comprehensive, and global analysis of transcriptomes (Nagalakshmi et al., 2008; Sultan et al., 2008; Wang et al., 2009). The RNA-seq approach has revealed a remarkable transcriptional heterogeneity of hippocampal CA1 pyramidal neurons and has provided a comprehensive database of transcription in hippocampal principal neurons, including the granule and mossy cells of the dentate gyrus and the pyramidal cells of the CA3, CA2 and CA1 areas (Cembrowski et al., 2016a,b). Therefore, investigating the potential transcriptional diversification within the developing hippocampus may provide a better understanding of neurodevelopmental disorders.

**Figure 1:**
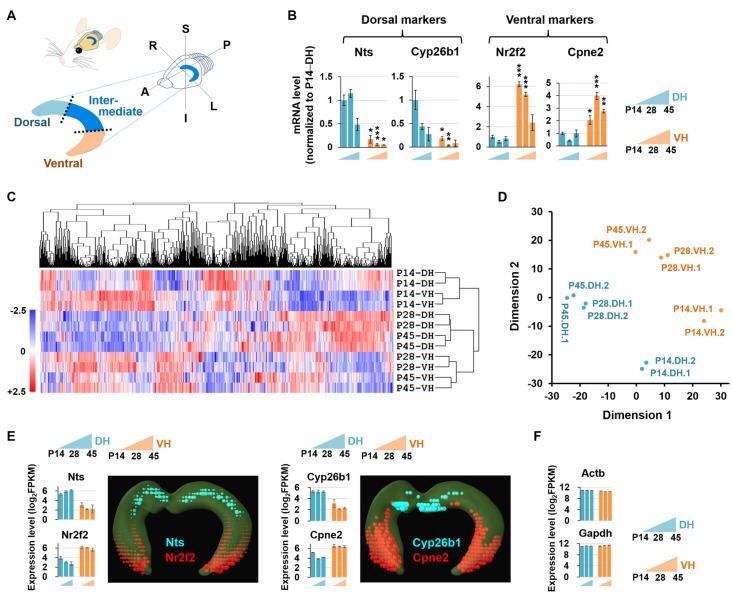
Experimental setup for RNA sequencing (RNA-seq)-based transcriptome analysis. **(A)** Diagram illustrating the dissection strategy of the dorsal and ventral hippocampus used in this study. A, anterior; P, posterior; S, superior; I, inferior; R, right; L, left. **(B)** Relative mRNA levels based on qRT-PCR results of the dorsal (Nts, Cyp26b1) and ventral (Nr2f2, Cpne2) markers in the developing hippocampus. *n* = 3–5 independent samples. Data represent mean ± SEM. *p < 0.05, **p < 0.005, ***p < 0.0005 compared with the corresponding DH at the same age, Student’s *t* test. Refer to Supplementary Table S5 for detailed statistics, including *n* number, *t*-value, degree of freedom and *p-value* for individual genes. **(C)** Hierarchical clustering analysis of RNA-seq data. Genes in duplicates are well clustered with one another. The values of log_2_FPKM were normalized to the value ranges from a minimum of −2.5 to a maximum of +2.5. **(D)** Multidimensional scaling plot for RNA-seq data. Dimension 1 and dimension 2 separate all 12 RNA-seq libraries based on the gene expression value. Euclidean distances were used to generate the plot. **(E,F)** Expression levels of the genes previously reported to be enriched in the dorsal (Nts, Cyp26b1) or ventral (Nr2f2, Cpne2) hippocampus **(E)** and evenly distributed throughout the hippocampus (Actb, Gapdh) **(F)**. Graphs indicate RNA-seq–based gene expression (rat brain). Data represent mean ± SEM. Images denote *in situ* hybridization–based gene expression (mouse brain) using ABA Brain Explorer 2. DH, dorsal hippocampus; VH, ventral hippocampus; P, postnatal day.

As the brain develops during early life, plasticity and maturation rates differ across the brain regions (Heim and Binder, 2012). Thus, the various regions and functional processes may be differentially sensitive to environmental insults at any given developmental time (Brydges et al., 2014). The hippocampus plays a crucial role in learning and memory processes and emotional behavior during development. The robust growth of the amygdala and hippocampus during the neonatal phase, that is, postnatal day (P) 0–P21, highlights the importance of neural and functional development. During the prepubertal phase (P21–P30), the cortical regions and the limbic system, including the hippocampus and amygdala, undergo structural and functional maturation. These limbic and cortical regions continue to mature during puberty and adolescence (P30–P60) and are actively involved in modulating hormonal stress reactivity in adulthood (Ulrich-Lai and Herman, 2009). Therefore, extensive knowledge of gene profiles during postnatal development of the hippocampus will provide gene set information that may vary with environmental perturbations or stress. In the present study, based on the developmental stages of rodents (Andersen and Teicher, 2008; Brenhouse and Andersen, 2011; Eiland and Romeo, 2013; Brydges et al., 2014), we examined three ages that correspond to three different postnatal developmental stages in rats, neonatal (P14), prepubertal (P28) and adolescence (P45) phases, using RNA-seq analysis to provide extensive information on the transcript profiles of the postnatally maturing hippocampus. This study is distinguished from previous RNA-seq studies as those studies explored only single developmental phases at P26–P35 (Cembrowski et al., 2016a,b). In addition, hippocampal subregions were divided into ventral and dorsal areas (Figure 1A) in the present study to identify the patterns of gene expression along the dorsal-ventral axis in the postnatally maturing hippocampus.

In the current study, we provide the first RNA-seq-based bioinformatic analysis of the developing dorsal and ventral hippocampi of rat brain at P14, P28 and P45. The RNA-seq data were verified by quantitative real-time polymerase chain reaction (qRT-PCR) and in part complemented by available *in situ* hybridization data from the mouse Allen Brain Atlas (ABA). Through the RNA-seq–based transcriptome analysis and qRT-PCR validation, we found that genes associated with long-term synaptic potentiation and long-term memory are strongly expressed in the dorsal hippocampus at P14 and P28 while genes associated with cholinergic synaptic transmission at P14, P28 and P45, and with the GABAergic synapse at P14, P28 and P45 are strongly expressed in the ventral hippocampus. In addition, we provide distinct segregations of transcripts associated with neural diseases along the dorsal-ventral axis of the hippocampus during postnatal development. Our analyses also revealed novel molecular markers enriched in the dorsal or ventral hippocampus. The extensive gene profiles provided in this study will serve as a reference to guide future investigations on the physiology and pathology of dorsal and ventral hippocampal functions.

## Materials and Methods

### Animals

All experimental protocols involving animals and their embryos were performed in accordance with the guidelines and regulations of the Korea Institute of Science and Technology. All experimental protocols were approved by the Institutional Animal Care and Use Committee at Korea Institute of Science and Technology (approval number: 2017-038).

### Sample Acquisition for RNA-seq and qRT-PCR

Sprague-Dawley rats aged P14, P28 and P45 were used for this study. Rats were anesthetized with halothane (2-bromo-2-chloro-1,1,1-trifluoroethane, Sigma Aldrich), and hippocampi were isolated. The hippocampus, including the dentate gyrus, CA3, CA2, CA1 and subiculum, was equally trisected to divide into a ventral, intermediate and dorsal portion, as shown in Figure 1A. Only the ventral and dorsal parts were collected for the study, and these ventral and dorsal hippocampal tissue lysates were used for total RNA isolation and subsequent RNA-seq and qRT-PCR analyses.

### RNA Extraction, cDNA Library Construction, RNA-seq and Data Analysis

RNA-seq was performed as previously described (Joe et al., 2017). Briefly, total RNA was extracted using an RNeasy kit (Qiagen, Valencia, CA, USA), and the RNA-seq library was then prepared using a TruSeq RNA Sample Prep kit (Illumina, San Diego, CA, USA). The sequencing was performed based on the Illumina NextSeq500 platform to generate 150 bp paired-end reads. The sequenced reads were mapped to the Rat genome (rn5) using TopHat 2, and the raw read counts were obtained using HT-seq (v.0.6.0; Anders et al., 2015). The edgeR (v. 3.12.1; McCarthy et al., 2012) was used to quantify gene expression from the RNA-seq data. Transcript expression levels were calculated as the fragments per kilobase of transcript per million fragments mapped (FPKM). The FPKM value of each gene was scaled via the trimmed mean of M-values (TMM) across all libraries for further analysis. In each analysis, differently expressed genes were selected through edgeR (v. 3.12.1). The criteria for determining a significantly altered gene were a fold change greater than 1.5 and a false discovery rate (FDR) less than 0.05. Heatmaps were constructed using MeV software (Howe et al., 2011). Statistical analyses and graph construction were performed using R (v. 3.3.0) and PYTHON (v. 2.7.6). The mapping statistics for RNA-seq were summarized in Supplementary Table S3.

### RNA-seq Data Access

RNA-seq data have been deposited in the National Center for Biotechnology Information Gene Expression Omnibus (GEO) under the accession number GSE96079; for a link for reviewers: https://www.ncbi.nlm.nih.gov/geo/query/acc.cgi?token=qlmjcoacxjonnmx&acc=GSE96079.

### Criteria for Categorizing Clusters

**Figure 2:**
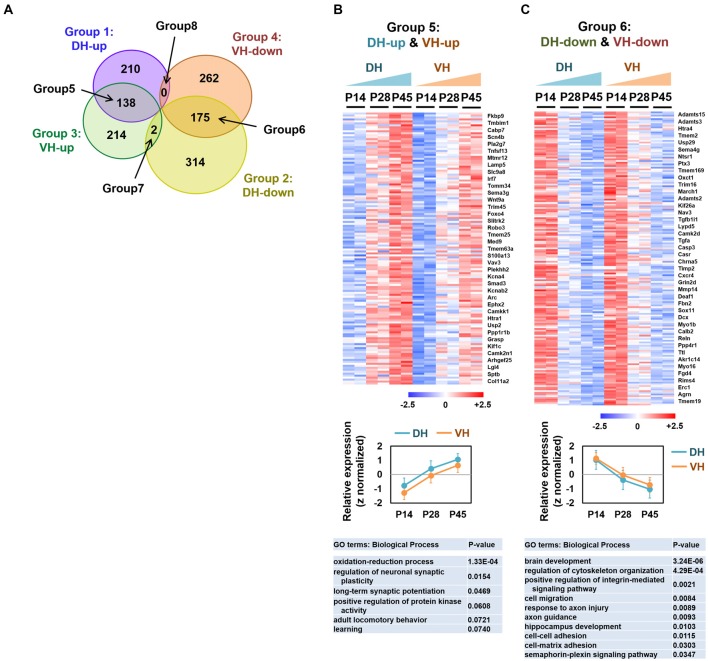
Analysis of the overlapping differentially expressed genes among groups 1–4. **(A)** Venn diagram illustrating the eight categories (groups 1–8) of the expression profiles in the dorsal or ventral hippocampus during postnatal development. **(B,C)** Heatmaps (upper), relative expression levels (middle) and gene ontology (GO) terms (biological process; lower) for the genes belonging to group 5 (DH-up and VH-up) **(B)** and to group 6 (DH-down and VH-down) **(C)**. Selected genes are represented on the right side of the heatmaps. The values of log_2_FPKM were normalized to the value ranges from a minimum of −2.5 to a maximum of +2.5. Graphs show the relative expression levels. Refer to Supplementary Figure S2.

For the classification of the up-regulated genes over development, significantly increased genes between P14 and P45 (P45/P14 > 1.5 and FDR < 0.05) were first selected for each dorsal and ventral hippocampus group. Among the genes, the up-regulated genes (P28/P14 > 1 and P45/P28 > 1) were selected by the comparison with the intermediate step, P28. For the classification of the down-regulated genes over development, significantly decreased genes between P14 and P45 (P14/P45 > 1.5, FDR < 0.05) were selected for each of the dorsal and ventral hippocampus group. Among the genes, the down-regulated genes (P14/P28 > 1 and P28/P45 > 1) were selected (Figure 2).

### Criteria for Dorsally or Ventrally Enriched Genes with RNA-seq Data

**Figure 3:**
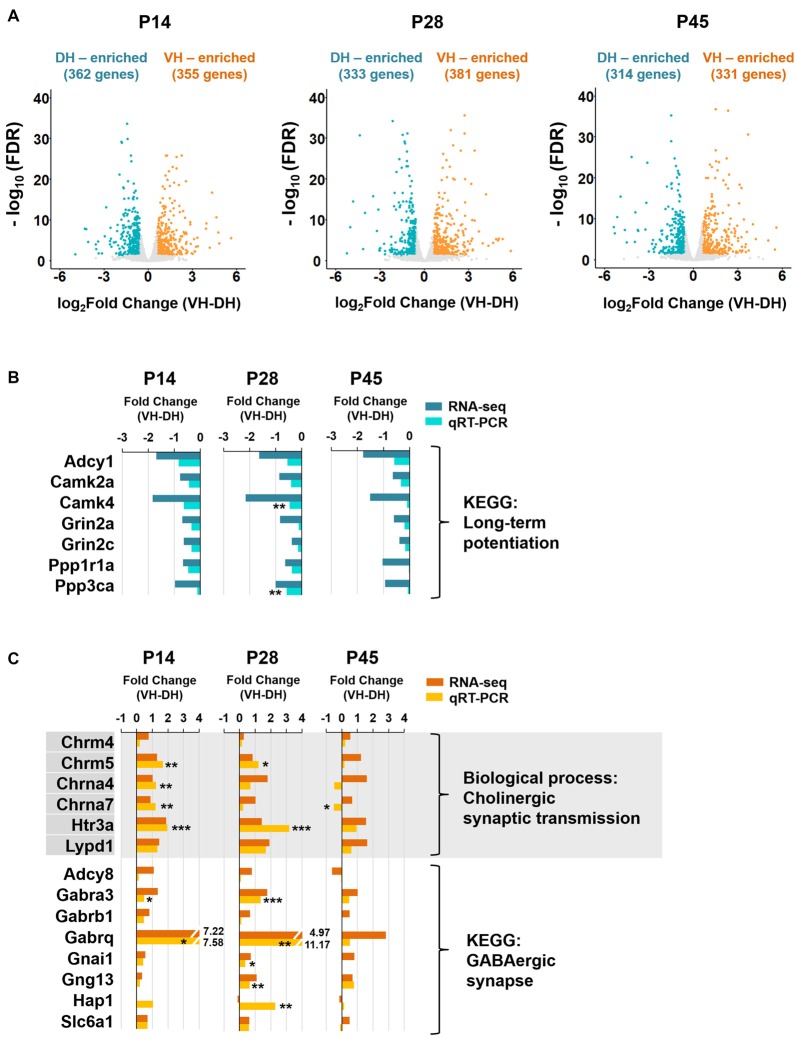
The long-term potentiation pathway is associated with the dorsal hippocampus, whereas cholinergic synaptic transmission and GABAergic synapse are associated with the ventral hippocampus during postnatal development.** (A)** Volcano plots for the dorsally or ventrally enriched genes in the hippocampus during postnatal development. Genes with a fold change of VH to DH ≥1.5 or ≤−1.5 were defined as ventrally or dorsally enriched genes, respectively, at individual developmental stages (P14, P28 and P45). Refer to Supplementary Table S1 for the full gene list at each age. DH, dorsal hippocampus; VH, ventral hippocampus. **(B)** qRT-PCR validations of the genes belonging to the long-term potentiation pathway associated with dorsally enriched gene groups at P14, P28 and P45 (Supplementary Figure S3B). RNA-seq and qRT-PCR values of each gene were compared at each developmental ages. *n* = 3–4 independent samples. **p < 0.005 for VH compared with DH, Student’s *t* test. Refer to Supplementary Table S5 for detailed statistics, including *n* number, *t*-value, degree of freedom and *p-value* for individual genes. **(C)** qRT-PCR validations of the genes belonging to the biological process of cholinergic synaptic transmission (Supplementary Figure S4A) and the KEGG pathway of GABAergic synapse (Supplementary Figure S4B) associated with ventrally enriched gene groups at P14, P28 and P45. RNA-seq and qRT-PCR values of each gene were compared at each developmental age, *n* = 3–5 independent samples *p < 0.05, **p < 0.005, ***p < 0.0005 for VH compared with DH, Student’s *t* test. Refer to Supplementary Table S5 for detailed statistics, including *n* number, *t*-value, degree of freedom and *p-value* for individual genes.

**Figure 4:**
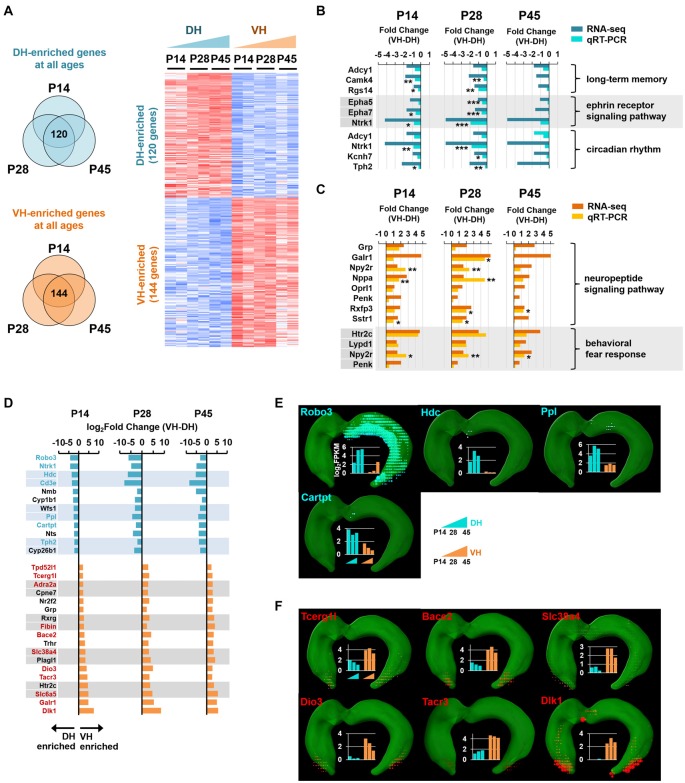
Analysis of enriched genes at all three developmental ages in the dorsal and ventral hippocampus.** (A)** Genes with a fold change of VH to DH ≥1.5 or ≤−1.5 at all developmental stages (P14, P28 and P45) were defined as ventrally or dorsally enriched genes, respectively. Heatmap for the dorsally or ventrally enriched genes. Refer to Supplementary Table S2 for the full gene list. **(B)** qRT-PCR validations for the genes belonging to the GO terms, long-term memory (Adcy1, Camk4, Rgs14), ephrin receptor signaling pathway (Epha5, Epha7, Ntrk1) and circadian rhythm (Adcy1, Ntrk1, Kcnh7, Tph2) targeted by the dorsally enriched genes at all three developmental ages (P14, P28, and P45) *n* = 3–5 independent samples. *p < 0.05, **p < 0.005, ***p < 0.0005 VH compared with DH, Student’s *t* test. Refer to Supplementary Table S5 for detailed statistics, including *n* number, *t*-value, degree of freedom and *p-value* for individual genes. Also refer to Supplementary Figure S5A. **(C)** qRT-PCR validations for the genes belonging to the GO terms, neuropeptide signaling pathway (Grp, Galr1, Npy2r, Nppa, Oprl1, Penk, Rxfp3, Sstr1), and behavioral fear response (Htr2c, Lypd1, Npy2r, Penk) targeted by the ventrally enriched genes at all three developmental ages. Refer to Supplementary Figure S5B. *n* = 3–5 independent samples. *p < 0.05, **p < 0.005 VH compared with DH, Student’s *t* test. Refer to Supplementary Table S5 for detailed statistics, including *n* number, *t*-value, degree of freedom and *p-value* for individual genes. **(D)** Genes with a fold change of VH to DH ≥4.0 or ≤−4.0 at all three developmental stages (P14, P28 and P45) are selected as ventrally or dorsally highly enriched genes, respectively. The gene names are presented on the left side of the graphs; genes in cyan and red font indicate newly identified potential markers for the dorsal and ventral hippocampus, respectively. **(E,F)** *In situ* hybridization data obtained using ABA Brain Explorer 2. Among the potential novel marker genes in **(D)**, only those that were validated by the ABA *in situ* hybridization data were presented as novel dorsal **(E)** and ventral **(F)** hippocampal markers. Graphs represent RNA-seq data; y-axis indicates the value of log_2_FPKM. Note that RNA-seq and qRT-PCR data were obtained from rat samples, whereas ABA *in situ* hybridization data were obtained from mouse samples.

**Figure 5:**
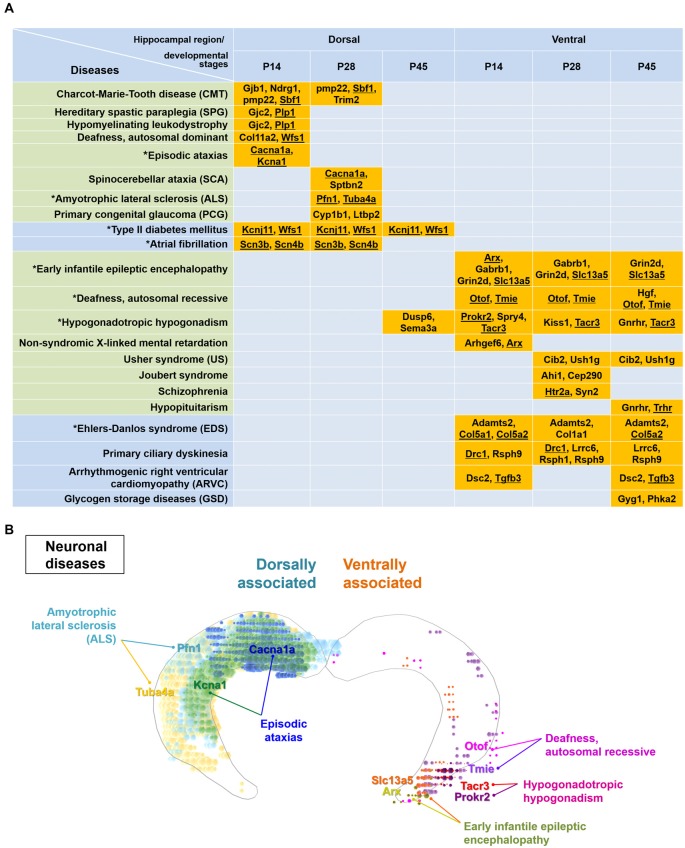
Particular disease-related genes are differentially expressed within either the dorsal or ventral hippocampus. **(A)** Genes enriched dorsally or ventrally in the hippocampus at each developmental age (Figure 3, Supplementary Table S1) were analyzed by the KEGG mapper program for disease search. Note that specific disease-related genes are distinctly segregated in the dorsal or ventral hippocampus, except for genes related to hypogonadotropic hypogonadism. Gene names are listed with the related diseases at individual developmental ages. Diseases presented in the green-shaded area are nervous system-related diseases. Underlined genes represent those enriched based on both the ABA *in situ* hybridization data and the RNA-seq data. Refer to Supplementary Figure S6 for the ABA *in situ* hybridization images for the underlined genes. Diseases marked with an asterisk were used to model the molecular domains related with specific diseases in **(B)** and Supplementary Figure S7. **(B)** Model for molecular domains related to neuronal diseases in the postnatally developing dorsal or ventral hippocampus. Based on the genes and their related diseases identified in this study in conjunction with the ABA *in situ* hybridization data, the spatial distribution of molecular domains related to the specific neuronal diseases was modeled. Only those molecules that share similar results between RNA-seq and ABA *in situ* hybridization analyses among the disease-related molecules in **(A)** were used for generating the molecular domains. Note that RNA-seq data were obtained from rat samples, whereas ABA* in situ* hybridization data were obtained from mouse samples. Refer to Supplementary Figure S7 for more information on the modeling of the spatial distribution of molecular domains related to non-neuronal diseases.

The dorsally or ventrally enriched genes were categorized by applying the same criteria (fold change 1.5, FDR < 0.05) for each time point (P14, P28 and P45), respectively (Figures 3–5). For highly enriched genes dorsally or ventrally (Figure 4D), the genes with a fold change of ventral hippocampus/dorsal hippocampus (VH/DH) ≥4.0 or ≤−4.0 at all developmental stages (P14, P28 and P45) were selected.

### Bioinformatic Analysis

The Database for Annotation, Visualization and Integrated Discovery (DAVID) Bioinformatics Resources v6.8^1^ (Huang da et al., 2009a,b; Joe et al., 2017) was applied for the GO analysis on the basis of three categories, including biological process, cellular component and molecular function, and also for the Kyoto Encyclopedia of Genes and Genomes (KEGG) enrichment analysis (Kanehisa and Goto, 2000; Kanehisa et al., 2014). For the disease analysis, rat genes belonging to the dorsally or ventrally enriched categories were converted to human homologous genes, which were then applied to the KEGG mapper tool^2^, a disease searching tool.

### Analysis of the ABA *in Situ* Hybridization Data

*In situ* hybridization data were obtained from the coronal or sagittal ABA images of the hippocampal regions, including Ammon’s horn and the dentate gyrus across the long hippocampal axis using mouse ABA Brain Explorer 2 (Lein et al., 2007; Lau et al., 2008). Dorsally enriched genes were marked in cyan, and ventrally enriched genes were marked in red. For all ABA images presented in this study, the intensity was ranged between 124 and >260, and the density was ranged between 0.0248 and >0.1, except for the following genes: Cyp26b1 (Figure 1E) and Cpne2 (Figure 1E) with intensities adjusted between 98 and >260; Tacr3 (Figure 4F, Supplementary Figure S6) with intensity adjusted between 79 and >166 and density adjusted between 0.00414 and >0.1; Kcna1 (Supplementary Figure S6) with intensity adjusted between 159 and >260; Otof (Supplementary Figure S6) with intensity adjusted between 79 and >154 and density adjusted between 0.0152 and >0.0779; Tmie (Supplementary Figure S6) with intensity adjusted between 108 and >154 and density adjusted between 0.0414 and >0.1; and Pfn1, Sbf1, Scn3b and Tuba4a (Supplementary Figure S6) with densities ranging between 0.0655 and >0.1 for clearer visualizations.

### qRT-PCR and Analysis

The isolated dorsal and ventral hippocampal tissues were homogenized using a Dounce Tissue Grinder (Wheaton), and subsequent total RNA extraction steps were followed as described in the manufacturer’s instructions. Briefly, total RNAs were purified from the isolated tissues using RNAiso Plus (TaKaRa, Japan). The cDNA was reverse transcribed from total RNA using a PrimeScript II 1st strand cDNA synthesis kit (TaKaRa, Japan). The qRT-PCR was performed using Power SYBR Green PCR Master Mix (Thermo Fisher Scientific). The reaction mixture contained 2 μL of cDNA corresponding to 100 ng of total RNA, 300 nM of each gene-specific primer, and 2× Power SYBR Green PCR Master Mix in a total volume of 20 μL. The cycling parameters of the StepOnePlus Real-Time PCR System (Applied Biosystems) were as follows: 95°C for 10 min, followed by 40 cycles of 95°C for 15 s and 60°C for 1 min. Relative expression levels were calculated according to the 2^−ΔΔCT^ algorithm that was internally normalized to the value of the glyceraldehyde 3-phosphate dehydrogenase (GAPDH) gene, which did not display differential expression among the samples in this study. Experiments were performed with three to five independent samples. The primer sets for the 40 genes used for qRT-PCR validations are listed in Supplementary Table S4.

To obtain a dorsally or ventrally enriched index, the relative expression level (2^−ΔΔCT^) of each sample (total of six samples: dorsal and ventral hippocampal samples at the three developmental stages represented on P14, P28 and P45) for each gene was normalized to that of the dorsal hippocampal sample at P14. Then, the normalized expression value in the dorsal hippocampus was subtracted from that in the ventral hippocampus at each developmental age. Genes with index values <0 or >0 were defined as enriched dorsally or ventrally, respectively.

## Results

### Experimental Strategy for RNA-seq Based Transcriptome Analysis of the Postnatal Dorsal and Ventral Hippocampus in Rats

The dorsal and ventral hippocampus have been shown to be functionally segregated (Moser and Moser, 1998; Bannerman et al., 2003; Lein et al., 2007; McHugh et al., 2011), and recent RNA-seq studies have provided comprehensive bioinformatic data supporting the notion of dorsal and ventral heterogeneity in the hippocampus (Zeisel et al., 2015; Cembrowski et al., 2016a,b). However, no RNA-seq–based bioinformatic study of the developing dorsal and ventral hippocampus has been reported. To investigate whether the dorsal and ventral hippocampus exhibit differential transcript profiles during postnatal development, and if so, whether the genes involved are related to biologically functional pathways and/or to neural diseases associated with these brain regions, we performed RNA-seq using 12 samples (in duplicate), which consisted of dorsal and ventral hippocampal tissues at three different postnatal developmental stages, P14, P28 and P45. We first tested whether the samples were appropriately prepared for this study by detecting established dorsal and ventral markers. The gene expression levels of the RNA-seq data were calculated as the fragments per kilobase of transcript per million fragments mapped (FPKM). Gene markers reported in previous studies of the dorsal (Nts, Cyp26b1) and ventral (Nr2f2, Cpne2) hippocampus (Leonardo et al., 2006; O’Reilly et al., 2015; Cembrowski et al., 2016a,b) were readily detected by qRT-PCR (Figure 1B) and RNA-seq (Figures 1E,F) with our experimental preparation and further cross-validated using the *in situ* hybridization results from the mouse ABA (Figure 1E). In addition, clustering analysis (Figure 1C) and multidimensional scaling (Figure 1D) of RNA-seq data showed sufficient clustering and similarity between the duplicate samples. These data together indicate that our samples are appropriately prepared for the study.

### Gene Expression Patterns in the Dorsal and Ventral Hippocampus

We categorized the RNA-seq–based transcript expression patterns into eight groups (Figure 2A): group 1 (or 2) for genes that increase (or decrease) expression over development in the dorsal hippocampus; group 3 (or 4) for genes that increase (or decrease) expression over development in the ventral hippocampus; group 5 for overlapping genes in groups 1 and 3; group 6 for overlapping genes in groups 2 and 4; group 7 for overlapping genes in groups 2 and 3; group 8 for overlapping genes in groups 1 and 4. To gain a better understanding of the gene expression properties in each group, we used a bioinformatic functional annotation tool, Database for Annotation, Visualization and Integrated Discovery (DAVID), to retrieve annotations from the gene ontology (GO) analysis based on the three categories, including biological process, cellular component, and molecular function (Ashburner et al., 2000) and from the Kyoto encyclopedia of genes and genomes (KEGG) pathway enrichment analysis (Kanehisa and Goto, 2000; Kanehisa et al., 2014) to identify enriched metabolic or signaling pathway gene patterns. Learning-related GO terms, such as learning or memory, long-term synaptic potentiation and learning were selected in groups 1 (DH-up), 3 (VH-up) and 4 (VH-down; Supplementary Figure S1). Genes belonging to groups 2 (DH-down) or 4 (VH-down) were associated with biological processes such as axon guidance, brain development, and/or neurogenesis (Supplementary Figure S1), indicating that transcript levels of genes belonging to those biological processes decrease over development, which is reasonable since axon guidance, brain development, and neurogenesis are predominant processes in early rather than later developmental stages.

Genes belonging to group 5 (DH-up and VH-up; Figures 2A,B) were associated with GO terms, such as oxidation-reduction process, regulation of neuronal synaptic plasticity, and long-term synaptic potentiation (Supplementary Figure S2A). Genes belonging to group 6 (DH-down and VH-down; Figures 2A,C) were associated with GO terms, such as brain development, cell migration, axon guidance, and response to axon injury, while also revealing their association with the KEGG pathways, such as extracellular matrix (ECM)-receptor interaction, axon guidance, microRNAs in cancer, and gap junctions (Supplementary Figure S2B). Only two genes (Mei1, Phkg2) were identified for group 7 (DH-down and VH-up), and no genes were identified for group 8 (DH-up and VH-down; Figure 2A, Supplementary Figure S1E).

### Analysis for Genes Enriched in Dorsal or Ventral Hippocampus at Different Postnatal Ages

We next identified genes with expression levels enriched in the dorsal or ventral hippocampus at the different developmental ages. Genes with fold changes in their FPKM value of ventral to dorsal hippocampus ≥1.5 or ≤−1.5 (FDR < 0.05) were defined as ventrally or dorsally enriched, respectively. A total of 362 and 355 genes at P14, 333 and 381 genes at P28, and 314 and 331 genes at P45 were selected as dorsally and ventrally enriched genes, respectively (Figure 3A). All genes belonging to dorsally or ventrally enriched groups at each developmental age are also listed in Supplementary Table S1.

We then conducted the GO enrichment and KEGG pathway analyses with the dorsally or ventrally enriched genes at individual developmental ages (Figure 3) in an attempt to identify the associated biological processes and pathways (Supplementary Figures S3, S4). Consistent with previous reports on dorsal hippocampus-dependent learning (Moser et al., 1993, 1995; McHugh et al., 2011), GO terms in biological processes, such as learning, long-term synaptic potentiation, and long-term memory at P14 and P28, and visual learning at P28, and the KEGG pathway long-term potentiation (Kouvaros and Papatheodoropoulos, 2016) at P14, P28 and P45 were associated with genes that were enriched in the dorsal hippocampus at individual ages (Supplementary Figure S3). The enrichment of genes belonging to the dorsal hippocampus-enriched KEGG pathway long-term potentiation were further validated by qRT-PCR (Figure 3B). Cholinergic synaptic transmission at P14, P28 and P45, the serotonin receptor signaling pathway at P14, P28 and P45, and morphine and nicotine addiction at P14, P28 and P45 were associated with genes enriched in the ventral hippocampus at each age (Supplementary Figure S4). Cholinergic synaptic transmission (Chrm4, Chrm5, Chrna4, Chrna7, Htr3a, Lypd1) and GABAergic synapse (Adcy8, Gabra3, Gabrb1, Gabrq, Gnai1, Gng13, Hap1, Slc6a1) associated with the ventrally enriched genes were further validated by qRT-PCR (Figure 3C), confirming the ventral hippocampus association with GABAergic and cholinergic synaptic transmission.

### Identification of Genes Enriched in the Dorsal or Ventral Hippocampus at all Postnatal Ages

We next selected the genes with fold changes in their FPKM value of ventral to dorsal hippocampus >1.5 (ventrally enriched; FDR < 0.05) or <−1.5 (dorsally enriched; FDR < 0.05) at all developmental stages (P14, P28 and P45). A total of 120 and 144 genes were selected as dorsally and ventrally enriched genes, respectively, at all ages (Figure 4A; see also Supplementary Table S2). Among these genes, those with a fold change >4.0 or <−4.0 at all developmental stages were selected as highly enriched genes in the ventral or dorsal hippocampus, respectively (Figure 4D). From these groups of genes, 7 (cyan font; Figure 4D) and 11 (red font; Figure 4D) genes were potentially identified as dorsal and ventral markers, respectively. Among the 7 cyan and 11 red font genes in Figure 4D, to further ensure the correct identification of markers, genes that were cross-validated with ABA *in situ* hybridization data were selected as novel dorsal (Figure 4E) and ventral (Figure 4F) markers for all developmental stages. A total of four genes as dorsal markers and six genes as ventral markers were discovered (Figures 4E,F). Further bioinformatic analysis revealed that biological processes, such as long-term memory (Adcy1, Camk4, Rgs14), the ephrin receptor signaling pathway (Epha5, Epha7, Ntrk1), and circadian rhythm (Adcy1, Ntrk1, Kcnh7, Tph2; Molero-Chamizo, 2013) are associated with the dorsal hippocampus (Supplementary Figure S5), and the genes associated with these processes were validated by qRT-PCR (Figure 4B). In addition, the ventral hippocampus was shown to be significantly associated with biological processes, such as neuropeptide signaling pathways (Grp, Galr1, Npy2r, Nppa, Oprl1, Penk, Rxfp3, Sstr1) and behavioral fear responses (Htr2c, Lypd1, Npy2r, Penk), and the genes belonging to these processes were validated by qRT-PCR (Figure 4C).

### Analysis of the Dorsally or Ventrally Enriched Genes at Individual Ages of the Postnatally Developing Hippocampus

Functional differentiation between the dorsal and ventral regions of the hippocampus has been experimentally and bioinformatically demonstrated in previous studies, strongly suggesting that hippocampal regions may have differential roles in distinct diseases. Indeed, schizophrenia, anxiety and depression have been associated with altered function of the ventral hippocampus (Tseng et al., 2009; Fanselow and Dong, 2010; Brooks et al., 2011; Small et al., 2011; O’Reilly et al., 2015).

Therefore, we further analyzed the genes dorsally or ventrally enriched in the postnatally developing hippocampus using the KEGG mapper tool, a disease searching tool (Kanehisa, 2013). Most diseases selected by the KEGG mapper tool, except hypogonadotropic hypogonadism, were distinctly segregated between the dorsal or ventral hippocampus (Figure 5A). Consistent with previous reports (Tseng et al., 2009; Brooks et al., 2011; O’Reilly et al., 2015), schizophrenia was associated with the genes ventrally enriched in the hippocampus (Figure 5A). We further identified that Charcot-Marie-Tooth disease, episodic ataxias spinocerebellar ataxia and amyotrophic lateral sclerosis are associated with gene profiles in the dorsal hippocampus, whereas early infantile epileptic encephalopathy, autosomal recessive deafness, Usher syndrome and Joubert syndrome are associated with those in the ventral hippocampus (Figure 5A). In addition to these neural diseases, type II diabetes mellitus and atrial fibrillation were associated with genes in the dorsal hippocampus while Ehlers-Danlos syndrome, primary ciliary dyskinesia and glycogen storage diseases were associated with genes in the ventral hippocampus (Figure 5A). We next cross-validated the expression patterns of the disease-related genes in Figure 5A with the ABA *in situ* hybridization data (Supplementary Figure S6). Based on the genes that were validated using the ABA data and our information on the associated diseases (Figure 5A), we generated a model of disease-related molecular domains specific to the dorsal and ventral hippocampus (Figure 5B, Supplementary Figure S7).

## Discussion

Our use of next generation RNA-seq technology has recapitulated previous studies and has expanded our understanding of the dorsal and ventral hippocampus by generating gene expression pattern information related to developmental stages. We performed a thorough analysis of the differentially expressed genes in the dorsal and ventral hippocampus at each or all developmental age(s) of P14, P28 and P45. We found that the pathways involving long-term potentiation and the glutamatergic synapse are associated with genes enriched in the dorsal hippocampus (Supplementary Figure S3B, Figure 3B), whereas the pathways involving the GABAergic synapse and serotonergic synapse are associated with genes enriched in the developing ventral hippocampus (Supplementary Figure S4B, Figure 3C). Furthermore, specific neural diseases were shown to be distinctively associated with gene expression in the dorsal or ventral hippocampus (Figure 5). Based on our bioinformatic analysis in conjunction with the ABA *in situ* hybridization data, we provide the molecular domains in the dorsal and ventral hippocampus associated with diseases (Figure 5B, Supplementary Figure S7). Thus, this study elucidates the physiological and pathological relationships among neural functions, hippocampal regions (dorsal vs. ventral) and postnatal developmental stages.

It has been reported that the anatomical structure of the hippocampus is vigorously formed during the late embryonic prenatal and early postnatal weeks (Iwata and Hevner, 2009; Lajud and Torner, 2015). The present study indicates that dorsal and ventral hippocampal gene expression profiles are clustered together at P14, and the clustering patterns become more similar to one another at later stages, that is, P28 and P45 (Figure 1C). These findings indicate that the hippocampus as a whole is transcriptionally more uniform and homogeneous at P14, which appeared to be the divergent time point for postnatal development defining the spatial-functional differentiation of the hippocampal subregions. Indeed, gene transcriptional differences between the dorsal and ventral hippocampus appeared at later stages of P28 and P45 (Figure 1C). Therefore, further investigation on the time-dependent transcriptional diversification of the hippocampus is warranted.

We identified several diseases differentially associated with gene enriched in the dorsal or ventral hippocampus during postnatal development (Figure 5). Although a limited number of genes were annotated as disease-associated genes, our results will provide a valuable reference for future investigations as they are consistent with those of previous studies reporting a disease-specific association with the ventral hippocampus, including for schizophrenia (Tseng et al., 2009; Brooks et al., 2011; O’Reilly et al., 2015). In addition, this analysis of disease-associated genes supports a differential regulation of a variety of candidates in the dorsal vs. ventral hippocampus across development. Therefore, it would be valuable to consider dorsal and ventral hippocampal development and function as well in the diseases that are not principally associated with the hippocampus.

Indeed, many of the diseases shown in Figure 5 are not principally related to hippocampal dysfunction, but each disease is directly or indirectly related to the learning and memory function of the hippocampus (Takeda et al., 2009; Liu et al., 2016; Shao and Stafstrom, 2016; Amado et al., 2017; Hadziselimovic et al., 2017). Early life seizures (early infantile epileptic encephalopathy) was reported to disrupt hippocampal place cell stability and thus the ability of animals to learn and remember a spatial location (Shao and Stafstrom, 2016). Episodic ataxia in patients appeared to be caused by misfiring of cerebellar Purkinje cells, and the associated seizures seemed to alter hippocampal firing (Rosenberg and Pascual, 2014). In addition, episodic ataxia symptoms triggered by fever were accompanied by a learning disorder that includes a low intelligence quotient as well as memory and executive function disturbances (Amado et al., 2017). Hypogonadotropic hypogonadism in cryptorchid boys with altered mini-puberty might affect neuronal genes important for long-term memory and learning-related intellectual abilities (Hadziselimovic et al., 2017). Acquired sensorineural hearing loss, such as deafness, impaired learning ability and cognitive performance by decreasing hippocampal neurogenesis in human and animal models (Liu et al., 2016). A hippocampal involvement in amyotrophic lateral sclerosis has also been reported by characterization of hippocampal degeneration in patients with this disease with or without memory deficits that is distinctive from Alzheimer’s disease (Takeda et al., 2009).

In the present study, we identified multiple genes that were dorsally or ventrally enriched at all ages examined (P14, P28 and P45) in the postnatally developing hippocampus (Figure 5) and proposed them as potential novel markers of the dorsal or ventral hippocampus. Many of the novel marker genes identified in the present study will serve as a platform to further reveal mechanisms specifically underlying dorsal or ventral hippocampal functions and their associated diseases. Dorsally or ventrally enriched markers at each individual age (Figure 3, Supplementary Table S1) and at all ages (Figure 4, Supplementary Table S2) were also identified, which will provide additional resources for the study of age-related neurodevelopmental diseases. In the hippocampal sample preparation we used for the RNA-seq analysis, hippocampal subregions, including the dentate gyrus, CA3, CA2, CA1 and subiculum, were divided into ventral, intermediate and dorsal areas (Figure 1A), and only the dorsal and ventral regions, but not the intermediate region, were analyzed. Although, because of the hippocampal sample preparation method, our findings from the RNA-seq data may appear to support the view of a dorsal-ventral dichotomy (Kjelstrup et al., 2008; Strange et al., 2014), we should take into consideration that at the individual gene level, there are various expression patterns, including gradual and step-wise changes or sharp transitions along the longitudinal axis (Thompson et al., 2008; Strange et al., 2014).

Although most genes enriched dorsally or ventrally as assessed by RNA-seq analysis showed a similar enrichment when assessed by qRT-PCR (Figures 3B,C, 4B,C), some genes, including Chrna4 at P45, Chrna7 at P45, Hap1 at P28 and P45 (Figure 3C), Thp2 at P45 (Figure 4B) and Galr1 at P45 (Figure 4C), displayed the opposite enrichment pattern. The reasons for this discrepancy between RNA-seq and qRT-PCR results are unknown but may have been caused by the individual variability among the rats in transcript levels and/or by the right and left hemisphere hippocampi possibly exerting different expression levels of the same genes. Further validation of the expression differences in larger cohorts would be required in future studies.

Enrichment patterns and transcript expression levels of specific genes selected from the rat RNA-seq data analysis were confirmed or cross-validated using the ABA mouse brain *in situ* hybridization data (Figures 4, 5). Many previous studies used ABA data to support their results from both mouse and non-mouse species (Ball-Rosen et al., 2007; Von Stetina et al., 2007; McHugh et al., 2008; Oldham et al., 2008; Sakakibara et al., 2008; Greene et al., 2009; Jones et al., 2009). It is certain that the use of the ABA data for non-mouse studies will likely facilitate new scientific findings, and we do be aware of species differences occurred between the mouse and rat in the present study. Indeed, when we applied the mouse ABA data to the rat RNA-seq data (Figures 4, 5), we excluded the genes selected by rat RNA-seq analysis when those results were not consistent with the mouse ABA data, generating data more stringent rather than intermingled from these two different species.

The use and interpretation of RNA-seq-based bioinformatic analysis have some limitations in that bioinformatic analysis can overlook important biological data caused by its reliance on known genes and known functions. In addition, biological pathways that have been more investigated than others accumulate more data and thus display stronger significance (Khatri and Drăghici, 2005; Huang da et al., 2009a,b; Tarca et al., 2013). Despite these limitations, RNA-seq is a promising high-throughput technology that has been recently applied to the field and has successfully provided precise information on the transcriptional dissection of molecular and cellular heterogeneity in various brain cell types and brain regions (Belgard et al., 2011; Pollen et al., 2014; Zhang et al., 2014; Usoskin et al., 2015; Zeisel et al., 2015; Cembrowski et al., 2016a,b; Joe et al., 2017).

The present study was the first to apply RNA-seq analyses to the developing dorsal and ventral hippocampus to investigate transcriptional differences along the dorsal and ventral axis during development, thereby providing a valuable resource for differentiating physiological and pathological functions within hippocampal regions and for future experimental elucidation of hippocampal functions. Certainly, RNA-seq combined with advanced genetic technologies will enable us to microdissect any brain region, including in a cell type-specific manner, which will further define molecular diversity throughout the brain and thereby foster structural and functional brain mapping to provide a better understanding of brain function.

## Author Contributions

A-RL sampled the dorsal and ventral hippocampus from rat brains and performed qRT-PCR, ABA analysis and RNA-seq analysis. EC sampled the dorsal and ventral hippocampus from rat brains. MK directed RNA-seq experiment, and J-HK performed RNA-seq analysis. MP conceived the study, designed the experiments, and performed bioinformatic analysis. A-RL and MP wrote the article with input from all authors.

## Conflict of Interest Statement

The authors declare that the research was conducted in the absence of any commercial or financial relationships that could be construed as a potential conflict of interest.

## Footnotes

^1^https://david.ncifcrf.gov

^2^http://www.genome.jp/kegg/tool/map_disease.html
