# Supplementary material for: Dorsal and Ventral Hippocampus Differentiate in Functional Pathways and Differentially Associate with Neurological Disease-Related Genes during Postnatal Development
Source: Front Mol Neurosci. 2017 Oct 16;10:331. doi: 10.3389/fnmol.2017.00331 (PMC5650623; doi:10.3389/fnmol.2017.00331)
Supplement: Supplementary file 3 [file Image_1.pdf]

Supplementary Material

Dorsal and ventral hippocampus differentiate in functional pathways and differentially associated with neurological disease-related genes during postnatal development

A-Ram Lee[1], Jong-Hwan Kim[2,3], Eunsil Cho[1,4], Mirang Kim[2,3] and Mikyoung Park[1,4]*

*** Correspondence:** Dr. Mikyoung Park, Korea Institute of Science and Technology, Seoul, South Korea, mikyoungpark7@gmail.com

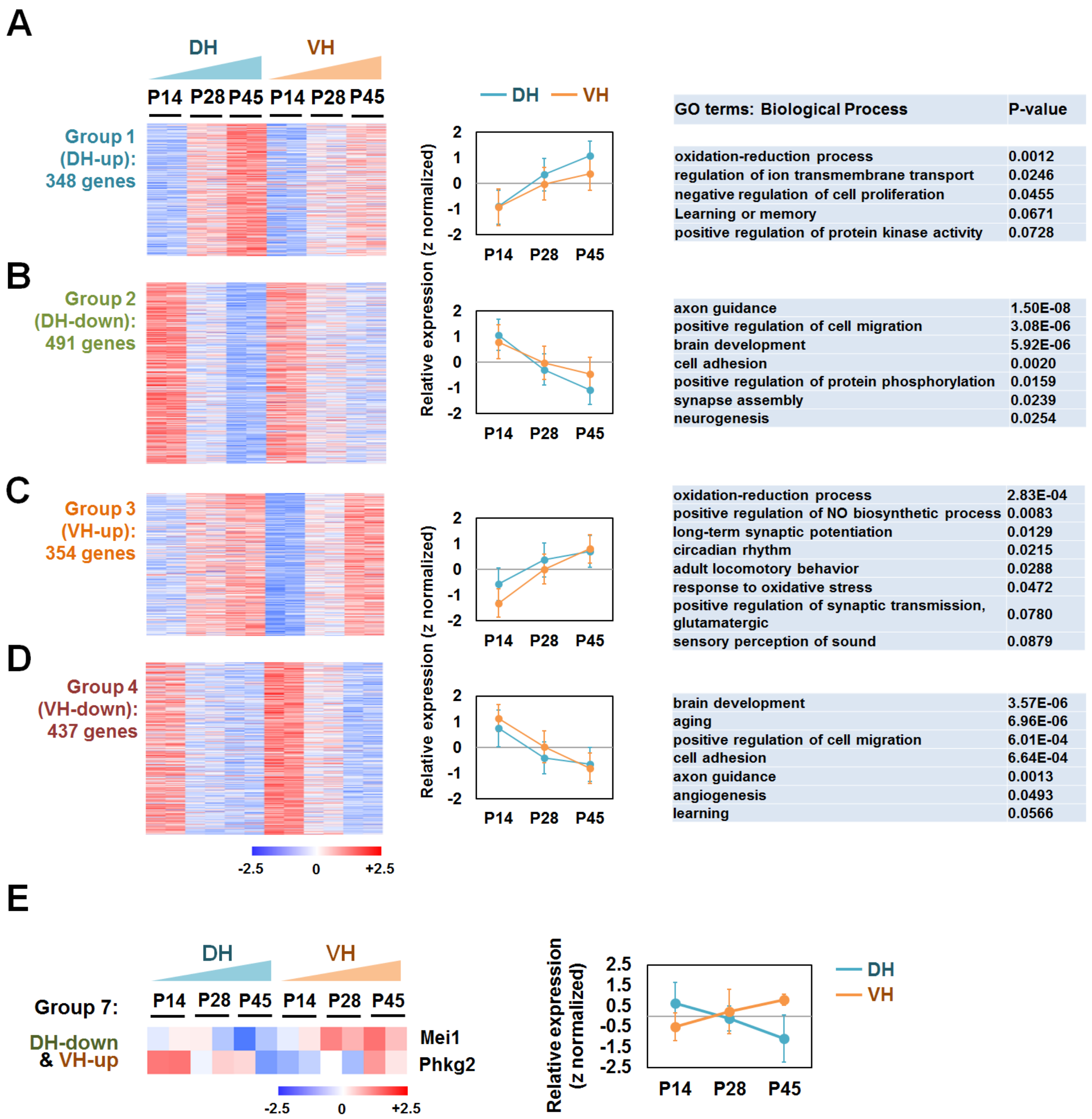

Supplementary Figure S1. Analysis of genes with expression levels increased or decreased over postnatal development in the dorsal or ventral hippocampus. (**A–D**) Heatmaps (left), relative expression levels (middle), and GO terms (biological process; right) for genes belonging to groups 1 to 4 (Figure 2A) are shown. In heatmaps, the values of $\log_2$FPKM were normalized to the value ranges from a minimum of –2.5 to a maximum of +2.5. In the graphs (middle), data represent means ± SEM. (**E**) Heatmaps for genes belonging to group 7 (DH-down and VH-up). Genes are presented on the right side of the heatmaps. The values of $\log_2$FPKM were normalized to the value ranges from a minimum of –2.5 to a maximum of +2.5. Graph shows the relative expression levels.

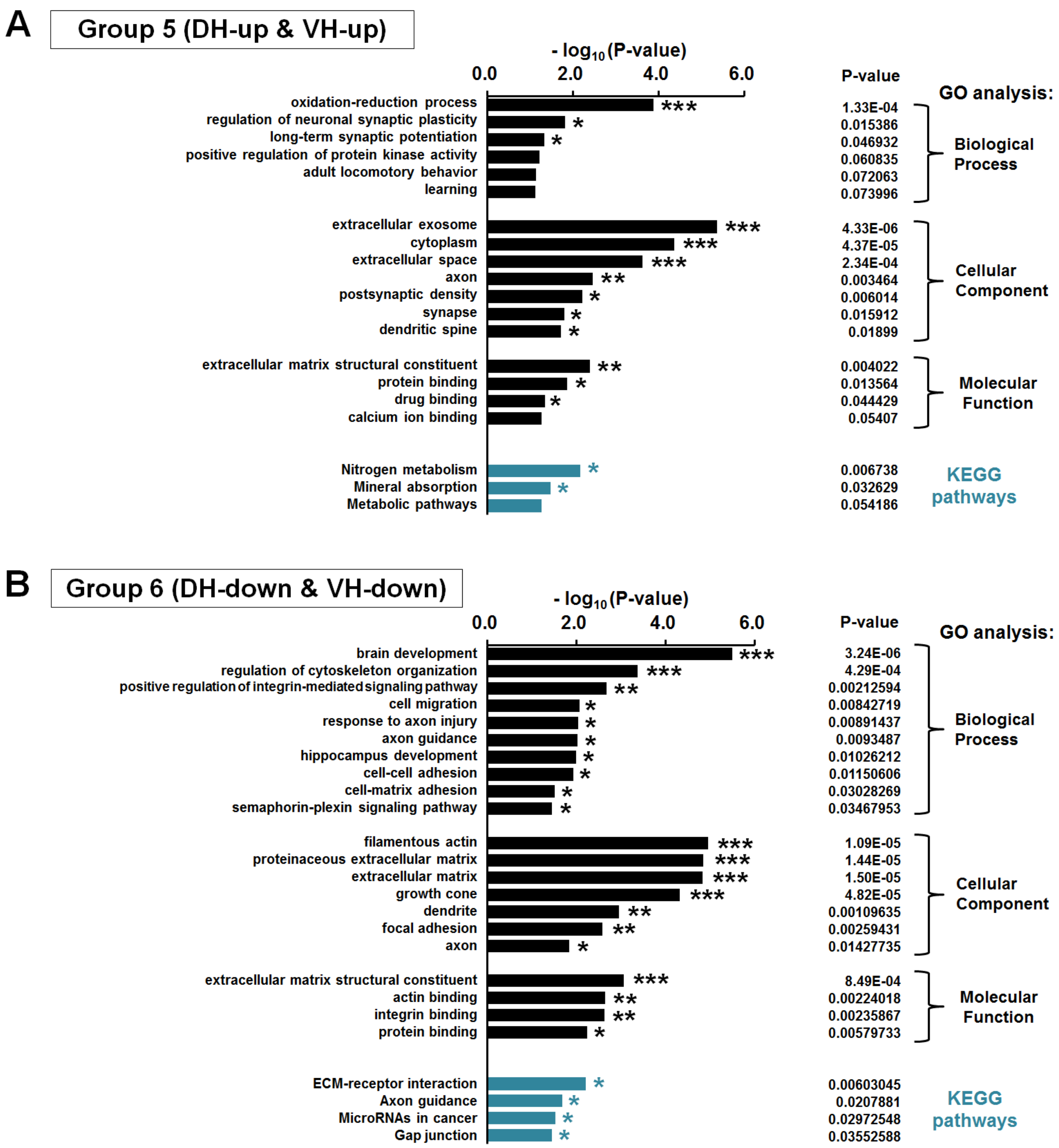

Supplementary Figure S2. GO and KEGG pathway enrichment analysis of genes belonging to group 5 (DH-up and VH-up) or 6 (DH-down and VH-down) in Figures 2B and 2C. GO and KEGG pathway analysis for the 166 differentially expressed genes in group 5 (DH-up and VH-up) (**A**) and for the 201 differentially expressed genes in group 6 (DH-down and VH-down) (**B**). *$p<0.05$, **$p<0.005$, ***$p<0.0005$, significantly enriched GO terms and KEGG pathways in DEGs. The X-axes indicate the enrichment of GO terms and KEGG pathways, and the Y-axes indicate the categories of GO terms and KEGG pathways.

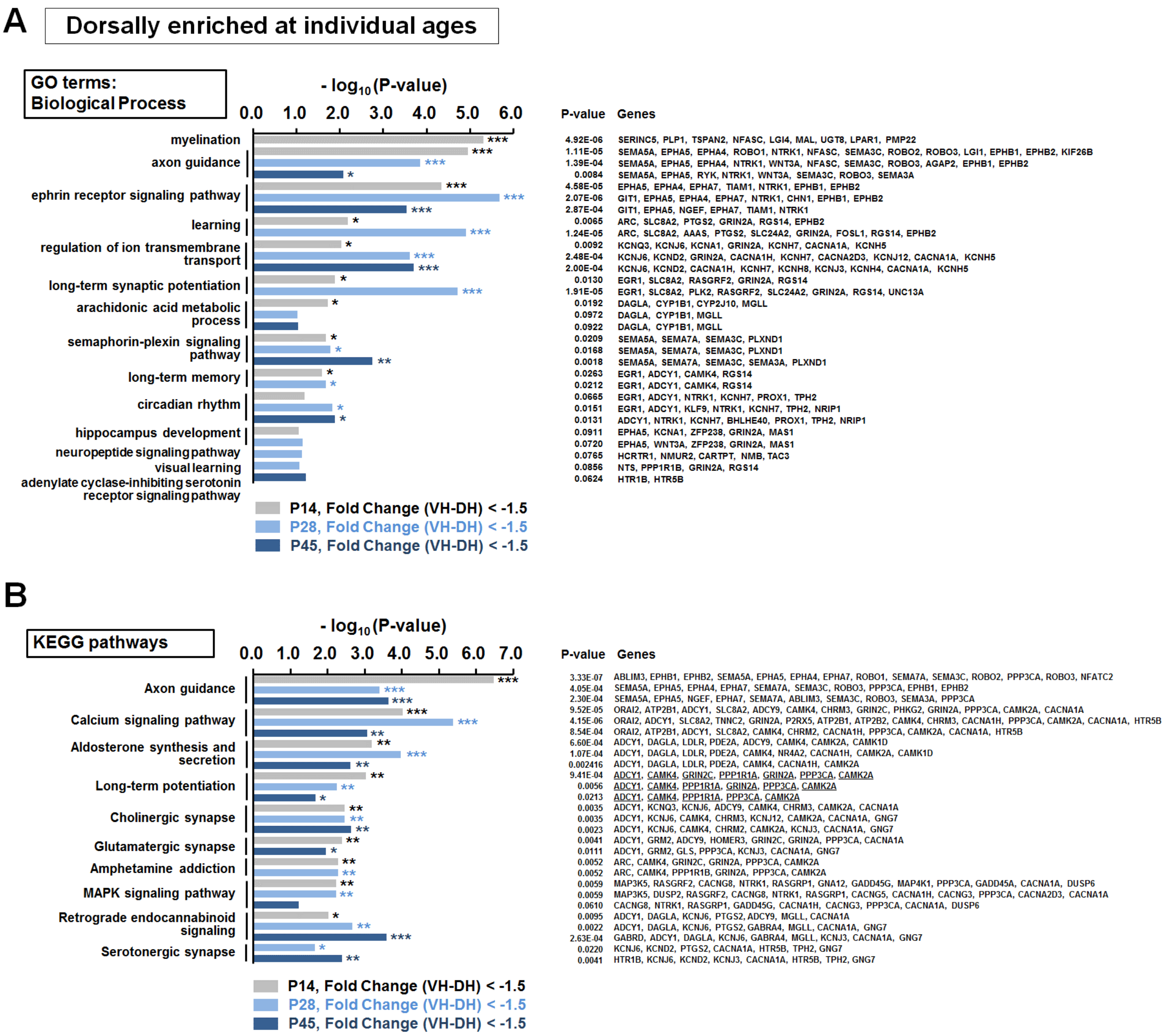

Supplementary Figure S3. GO term and KEGG pathway analysis of dorsally enriched genes at individual ages in Figure 3. GO terms (**A**) and KEGG pathways (**B**) targeted by dorsally enriched genes at individual developmental stages (P14, P28, and P45) were analyzed. Gene list and P-values corresponding to each term and pathway are shown. qRT-PCR–validated genes are underlined. *p<0.05, **p<0.005, ***p<0.0005, significantly enriched GO terms and KEGG pathways. The X-axes indicate the enrichment of GO terms and KEGG pathways, and the Y-axes indicate the categories of GO terms and KEGG pathways.

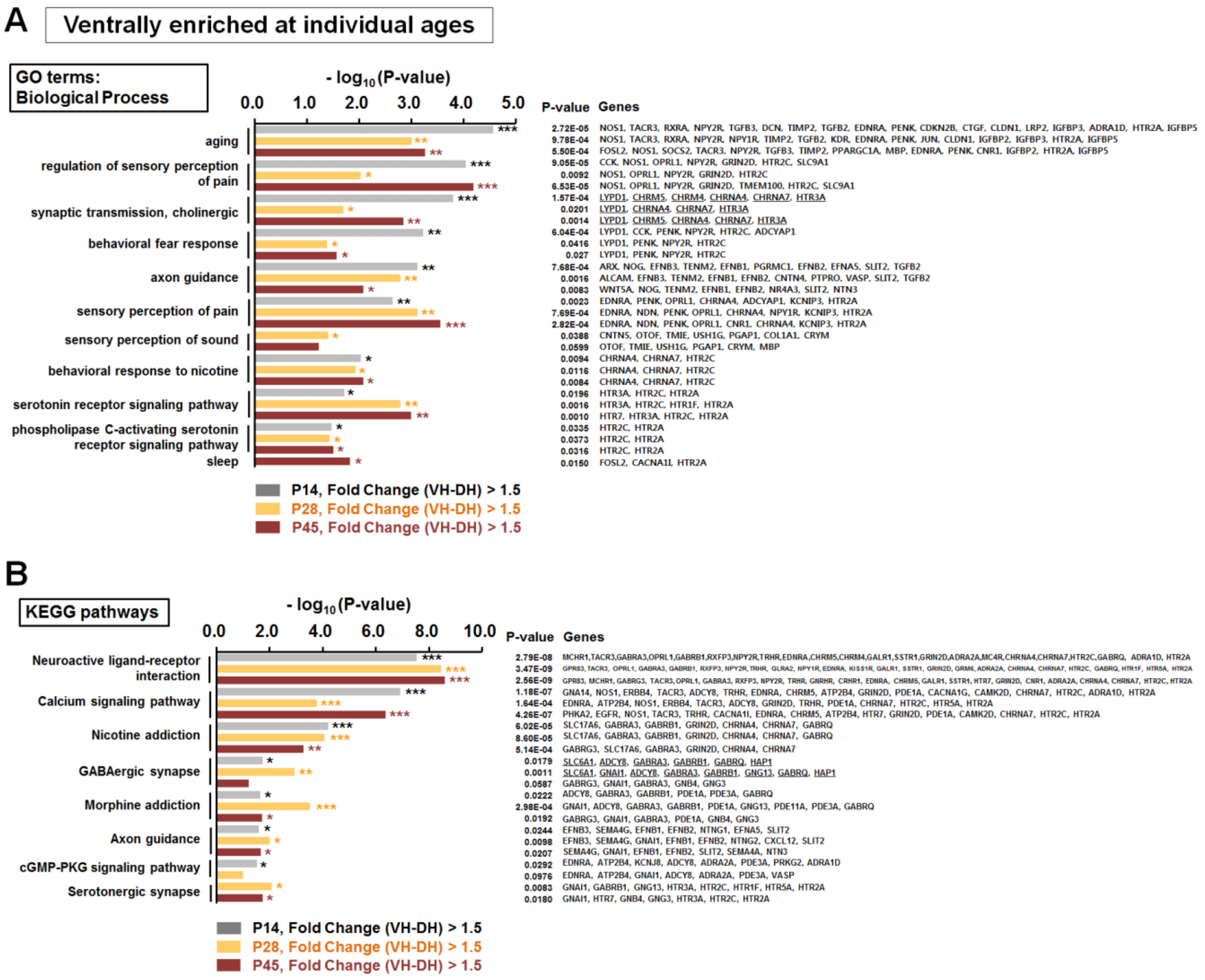

Supplementary Figure S4. GO term and KEGG pathway analysis of ventrally enriched genes at individual ages in Figure 3. GO terms (**A**) and KEGG pathways (**B**) targeted by ventrally enriched genes at individual developmental stages (P14, P28, and P45) were analyzed. Gene list and P-values corresponding to each term and pathway are shown. qRT-PCR validated genes are underlined. *p<0.05, **p<0.005, ***p<0.0005, significantly enriched GO terms and KEGG pathway. The X-axes indicate the enrichment of GO terms and KEGG pathways, and the Y-axes indicate the categories of GO terms and KEGG pathways.

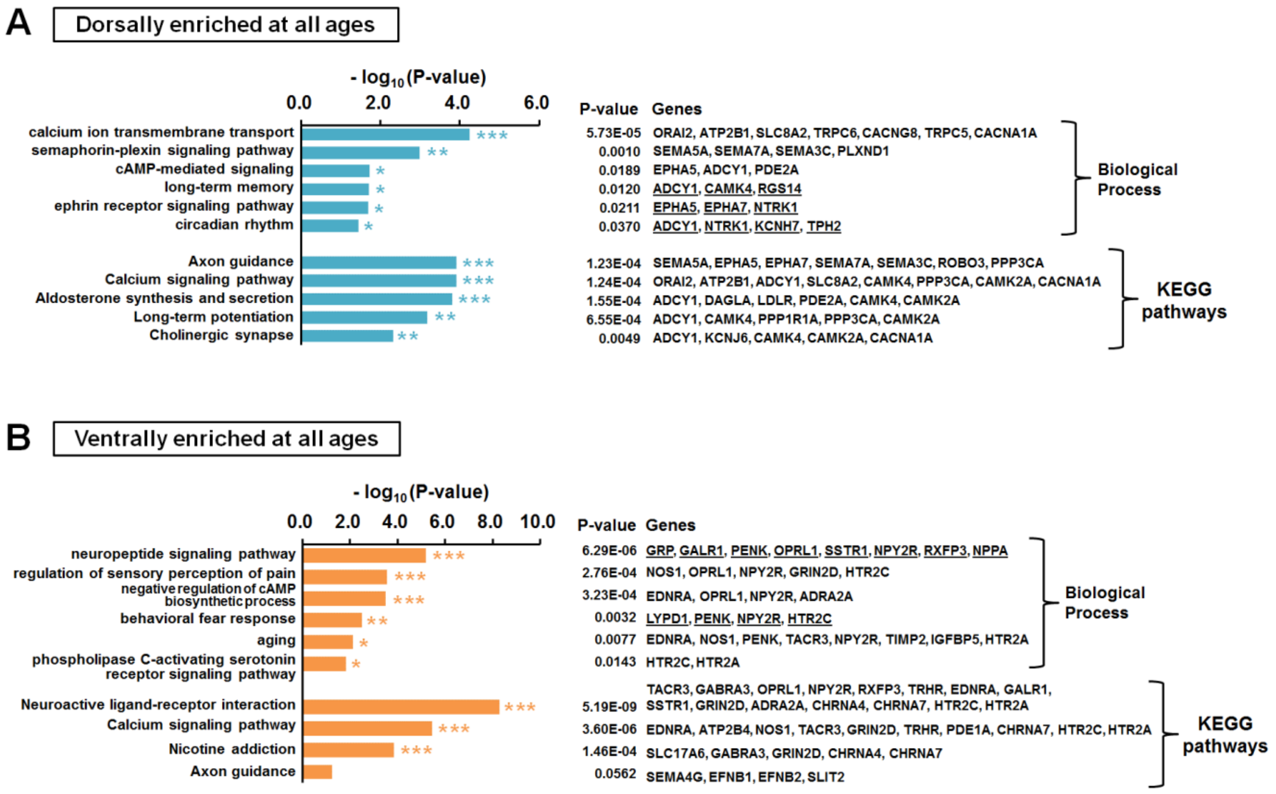

Supplementary Figure S5. GO term and KEGG pathway analysis of the genes in Figure 4. GO terms and KEGG pathways targeted by dorsally (**A**) or ventrally (**B**) enriched genes at all developmental stages (P14, P28, and P45) were analyzed. Gene list and P-values corresponding to each term and pathway are shown. qRT-PCR validated genes are underlined. *p<0.05, **p<0.005, ***p<0.0005, significantly enriched GO terms and KEGG pathways. The X-axes indicate the enrichment of GO terms and KEGG pathways, and the Y-axes indicate the categories of GO terms and KEGG pathways.

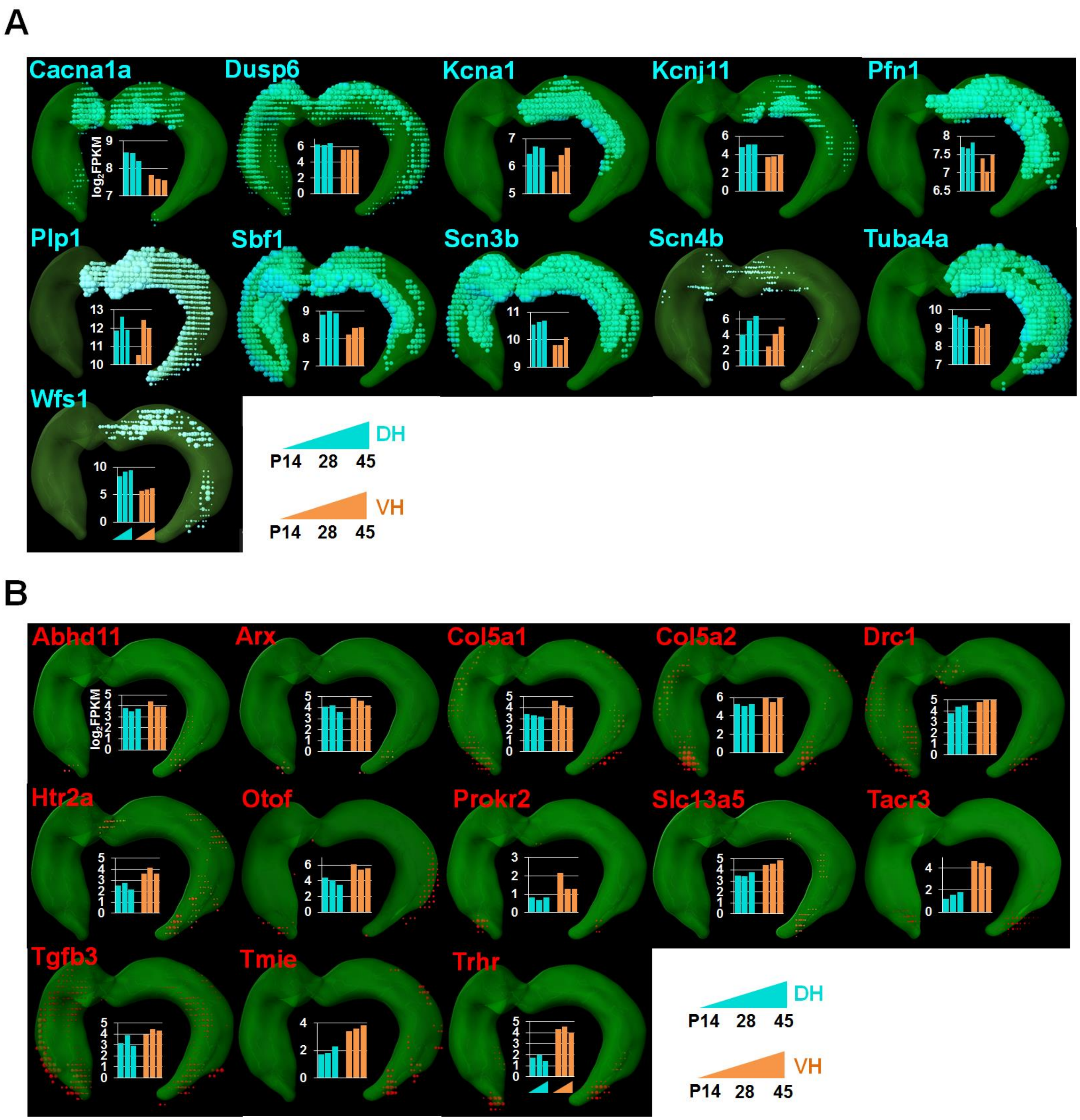

Supplementary Figure S6. The ABA *in situ* hybridization images for the disease-related genes in Figure 5. Among the genes related to the diseases listed in Figure 5, only those showing consistency in expression patterns (dorsally or ventrally enriched) between RNA-seq and ABA *in situ* hybridization data were presented. Transcript expression levels of the disease-related dorsally enriched genes (**A**) or ventrally enriched genes (**B**) were shown by RNA-seq (graphs) and *in situ* hybridization (images). Note that RNA-seq data were obtained from rat samples, whereas ABA *in situ* hybridization data were obtained from mouse samples.

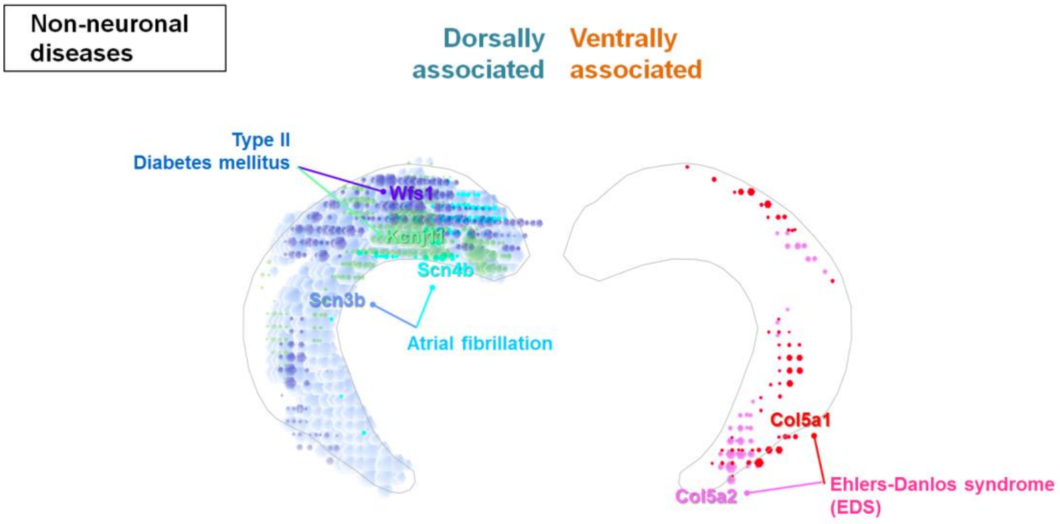

Supplementary Figure S7. Model for molecular domains related to non-neuronal diseases in the postnatally developing dorsal or ventral hippocampus. Based on our identification of the genes and their related diseases in conjunction with the ABA *in situ* hybridization data, the spatial distribution of molecular domains related to the specific non-neuronal diseases was modeled. Only those molecules that share similar results between RNA-seq and ABA *in situ* hybridization analyses among the disease-related molecules in Figure 5A were selected. Note that RNA-seq data were obtained from rat samples, whereas ABA in situ hybridization data were obtained from mouse samples.

Supplementary Table S1. Full gene list of dorsally or ventrally enriched group at individual developmental ages in Figure 3.

Separately uploaded as an excel file.

Supplementary Table S2. Full gene list of dorsally or ventrally enriched group at all developmental ages in Figure 4.

Separately uploaded as an excel file.

Supplementary Table S3. Mapping statistics.

Samples	Output reads	Uniquely mapped pairs	Mapping rate (%)
P14-DH-1	27,681,476	17,525,597	63.30
P14-DH-2	22,492,264	14,881,853	66.20
P14-VH-1	27,023,612	17,924,402	66.30
P14-VH-2	20,816,034	13,626,603	65.50
P28-DH-1	19,592,366	12,943,706	66.10
P28-DH-2	15,940,826	10,509,726	65.90
P28-VH-1	22,519,926	14,701,036	65.30
P28-VH-2	23,021,816	14,859,907	64.50
P45-DH-1	24,415,898	16,112,912	66.00
P45-DH-2	27,123,716	17,594,199	64.90
P45-VH-1	23,088,756	15,154,497	65.60
P45-VH-2	23,128,946	15,244,652	65.90

Supplementary Table S4. Primer sets used for quantitative real-time PCR.

Gene	Forward primer (5' to 3')	Reverse primer (5' to 3')	*Acc. No.
Adcy1	GCGGCTGGAAGATGAGAATG	CATTGTCGTGCCTCTGGATG	NM_001107239.1
Adcy8	TGCTGCAAGTCACACTTCAA	CTGATGAAGATGCCTGCTGT	NM_017142.1
Camk2a	GGGACACCTGGATACCTCTC	AGAATGGGGGATACCCAACC	NM_012920.1
Camk4	GAACCTCGTCCCGGATTACT	CTTCTGGGTCCCCTTCTGTT	NM_012727.3
Chrm4	GTCACCAAACCCCTCACCTA	CAGAACAAGATGGCAGGAGC	NM_031547.1
Chrm5	ACCCCATCTGCTATGCTCTC	AGGGTAGCTTGCTGTTTCCT	NM_017362.4
Chrna4	TCTATGACGGAAGGGTGCAG	ATCTTGGCCTTGTCGTAGGT	NM_024354.1
Chrna7	TGGCTCTGCTGGTATTCTTG	ATGATCTCAGCCACAAGCAG	NM_012832.3
Cpne2	CTGGAAGGTGTCCCATGAGT	CATAGAAGCGAATGTGGGGC	NM_001256466.1
Cyp26b1	GCTCATCGGAGAGACTGGT	GCCCCAGTAAGTGTGTCTTGA	NM_181087.2
Epha5	ACCACACCAGTGTTTGCAG	GAGGAAGCCGATCATCACTG	NM_001169137.2
Epha7	CCAGGCACCAAAACCTACAT	ACGACCACTGCAAACTTCTC	NM_134331.1
Gabra3	AGTGATTGCTTCCCCCAAGA	TATGGCAAAGAGCACAGGGA	NM_017069.3
Gabrb1	GGTGTCCAAGAAGGTGGAAT	ATCAGTGTGGAGGGCATGTA	NM_012956.1
Gabrq	GTGCAAGAAGCAAGTTGGGA	CCCCTTTGCGTTGTTCATCA	NM_031733.1
Galr1	ACAGCAACCAAACCTTCTGC	AACCTTGGCATAGCAAAAGCA	NM_012958.3
Gnai1	AGAGTCCCCTCACGATATGC	CGCAAGTGAAGTGGGTGTAAA	NM_013145.1
Gng13	GGAGATGTCGTCCAAGACCA	TACACTTGGCCTTCTCCACC	NM_001135918.1
Grin2a	CATCAATGAGGAGCGTTCGG	AAGGGTTCGAGGAAAGCAGA	NM_012573.3
Grin2c	CCGTGTGGGTGATGATGTTC	GGTTTCTTGCCCTTGGTGAG	NM_012575.3
Grp	CACTGGGCTGTAGGACACTT	GAGGAGGCCAAGCAAATTCC	NM_133570.5
Hap1	GTATGTCCTCCAGCAACTGTC	GTTGATGATCGGTAGCTTGTCC	NM_024133.2
Htr2c	ACTGGCCAGCACTTTCAATC	TCCCACCAGCATATCAGCAA	NM_012765.3
Htr3a	TAAGCAGGATTTACAGCGCC	TGGCTGAGCAGTCATCAGT	NM_024394.2
Kcnh7	GTGATGCATCGTCAGAGCTT	ACCCAAGATCCCTACAGTGC	NM_131912.1
Lypd1	GGGTTCTCGGCATCGCA	CGTTCACGGTGCAATTGACTA	NM_001007727.1
Nppa	CCTCCGATAGATCTGCCCTC	CTGTCAATCCTACCCCCGAA	NM_012612.2
Npy2r	AGCCTTTCCACCCTGCTAAT	GCCTTCGCTGATGGTAATGG	NM_023968.1
Nr2f2	AGGCCATAGTCCTGTTCACC	GTTGGGGTACTGGCTCCTAA	NM_080778.2
Ntrk1	TTCAGCACCGAGAGTGATGT	ATCGCCTCAGTGTTGGAGAG	NM_021589.1
Nts	GATGACGACCTTGTTGCGAA	ATCTCCCAGTGTTGAAAGGC	NM_001102381.1
Oprl1	GCACTTTTACTCTGACCGCC	ACAGGAACACCAACCACTGA	NM_001318948.1
Penk	CTGCCTCCTGGCTACAGTG	GCTGCCCTTCACATTCGAG	NM_017139.1
Ppp1r1a	GACCAGTCATCCCCAGAAGT	TCTGGAGCTCTTTCATGGTG	NM_022676.3
Ppp3ca	GGCGATTGATCCCAAGTTGT	CGAGGCTTCCCATCGTTATC	NM_017041.1
Rgs14	GAGCGTTTCCAACAGATCCC	CTGTCGGTCGATGTTCACTG	NM_053764.1
Rxfp3	GATAGCTTCGCTGCCCAATG	GGTACAAACCCAGCCAGAAC	NM_001008310.1
Slc6a1	CCTGGGCCATCTACTACCTG	CACTGGTCATGTTGGTGGTG	NM_024371.2
Sstr1	TCAGCTGGATGGATAACGCT	AAACGCCTCCAGATTCCAGA	NM_012719.2
Tph2	TAGAGGATGTGCCGTGGTTC	TCCTTGAATCCTGGGTGGTC	NM_173839.2

*Acc. No. indicates gene access number.

Supplementary Table S5. Statistics on quantitative real-time PCR data in Figures 1, 3, and 4.

	GO terms/ KEGG pathways	Genes		Student's *t* test			
				N number (P14, P28, P45)	t-value (P14/P28/P45)	degree of freedom (P14/P28/P45)	p-value (P14/P28/P45)
Figure 1B		Nts	DH	3, 3, 4	4.46/12.7/2.76	4/4/4	0.011/0.0002/0.040
			VH	3, 3, 3			
		Cyp26b1	DH	3, 3, 3	4.79/6.03/1.23	4/4/4	0.0087/0.0038/0.29
			VH	3, 3, 3			
		Nr2f2	DH	5, 5, 4	-17.7/-20/-2.28	6/6/5	<0.0001/<0.0001/0.071
			VH	3, 3, 3			
		Cpne2	DH	3, 4, 4	-2.99/-14.5/-4.91	5/5/5	0.030/<0.0001/0.0045
			VH	4, 3, 3			
Figure 3B	long-term potentiation	Adcy1	DH	3, 3, 3	1.96/1.88/1.26	5/5/4	0.11/0.12/0.28
			VH	4, 4, 3			
		Camk2a	DH	4, 4, 4	1.32/1.04/0.853	14/14/11 (in duplicate except VH-P45)	0.21/0.31/0.41
			VH	4, 4, 4			
		Camk4	DH	4, 4, 4	2.33/5.44/0.608	6/6/6	0.058/0.0016/0.57
			VH	4, 4, 4			
		Grin2a	DH	4, 4, 4	1.06/0.369/0.808	14/14/14 (in duplicate)	0.31/0.72/0.43
			VH	4, 4, 4			
		Grin2c	DH	4, 4, 4	1.67/0.692/0.981	14/14/14 (in duplicate)	0.12/0.50/0.34
			VH	4, 4, 4			
		Ppp1r1a	DH	4, 4, 4	1.33/1.97/0.147	14/14/14 (in duplicate)	0.20/0.069/0.89
			VH	4, 4, 4			
		Ppp3ca	DH	4, 4, 4	0.657/3.44/0.366	14/13/11 (in duplicate except DH-P28, P45 & VH-P45)	0.52/0.0044/0.72
			VH	4, 4, 4			
Figure 3C	cholinergic synaptic transmission	Chrm4	DH	4, 4, 4	-1.11/-1.59/-1.54	13/11/12 (in duplicate except DH-P28 & VH-P14, P28)	0.29/0.14/0.15
			VH	4, 4, 3			
		Chrm5	DH	5, 5, 5	-4.92/-3.88/-0.480	7/7/6	0.0017/0.0061/0.65
			VH	4, 4, 3			
		Chrna4	DH	3, 3, 3	-6.51/-0.991/0.938	4/4/4	0.0029/0.38/0.40
			VH	3, 3, 3			
		Chrna7	DH	4, 4, 4	-4.21/-0.519/3.26	12/13/12 (in duplicate except DH-P45 & VH-all ages)	0.0012/0.61/0.0068
			VH	4, 4, 4			
		Htr3a	DH	5, 5, 5	-6.93/-16.8/-1.57	8/8/6	0.0001/<0.0001/0.17
			VH	5, 5, 3			
		Lypd1	DH	3, 3, 3	-1.05/-1.34/-0.744	4/4/4	0.35/0.25/0.50
			VH	3, 3, 3			
	GABAergic synapse	Adcy8	DH	4, 3, 3	-0.492/-0.450/0.210	6/5/4	0.64/0.67/0.84
			VH	4, 4, 3			
		Gabra3	DH	4, 4, 3	-3.51/-8.37/-1.10	5/5/4	0.017/0.0004/0.33
			VH	3, 3, 3			
		Gabrb1	DH	4, 4, 4	-1.54/-0.663/-0.327	6/6/6	0.17/0.53/0.75
			VH	4, 4, 4			
		Gabrq	DH	4, 4, 4	-3.15/-6.43/-0.505	6/6/6	0.020/0.0007/0.63
			VH	4, 4, 4			
		Gnai1	DH	3, 3, 4	-1.06/-4.52/0.0159	4/4/5	0.35/0.011/0.99
			VH	3, 3, 3			
		Gng13	DH	3, 3, 3	-1.74/-6.59/-2.48	4/4/4	0.16/0.0027/0.068
			VH	3, 3, 3			
		Hap1	DH	3, 4, 3	-2.50/-7.34/-0.347	5/5/4	0.054/0.0007/0.75
			VH	4, 3, 3			
		Slc6a1	DH	3, 4, 3	-0.933/-1.99/0.393	4/5/4	0.40/0.10/0.71
			VH	3, 3, 3			

**Figure 4B**	long-term memory	Adcy1	DH	4, 4, 4	1.19/1.28/1.12	6/6/6	0.28/0.25/0.31
			VH	4, 4, 4			
		Camk4	DH	4, 5, 4	4.33/5.55/0.463	7/7/6	0.0035/0.0009/0.66
			VH	5, 4, 4			
		Rgs14	DH	4, 4, 4	2.53/3.83/0.888	14/14/12 (in duplicate except DH-P45 & VH-P45)	0.024/0.0018/0.39
			VH	4, 4, 4			
	ephrin receptor signaling pathway	Epha5	DH	3, 4, 4	1.04/7.56/0.871	5/6/6	0.35/0.0003/0.42
			VH	4, 4, 4			
		Epha7	DH	3, 4, 3	3.34/7.44/1.45	4/6/4	0.029/0.0003/0.22
			VH	3, 4, 3			
		Ntrk1	DH	4, 4, 3	3.95/9.07/1.43	6/6/4	0.0076/0.0001/0.23
			VH	4, 4, 3			
	Circadian rhythm	Adcy1	DH	4, 4, 4	1.36/1.31/1.07	6/6/6	0.22/0.24/0.33
			VH	4, 4, 4			
		Ntrk1	DH	4, 4, 3	6.92/18.3/1.47	6/6/6	0.0005/<0.0001/0.19
			VH	4, 4, 5			
		Kcnh7	DH	4, 4, 4	2.05/3.12/1.82	14/14/10 (in duplicate except VH-P45)	0.059/0.0075/0.098
			VH	4, 4, 4			
		Tph2	DH	3, 4, 3	2.64/4.72/-1.68	5/6/4	0.046/0.0033/0.17
			VH	4, 4, 3			
**Figure 4C**	neuropeptide signaling pathway	Grp	DH	3, 3, 3	-1.97/-0.865/-1.13	5/5/4	0.11/0.43/0.32
			VH	4, 4, 3			
		Galr1	DH	4, 4, 3	-1.32/-3.42/0.659	6/5/4	0.24/0.019/0.55
			VH	4, 3, 3			
		Npy2r	DH	4, 4, 3	-5.64/-4.88/-0.902	5/6/4	0.0024/0.0028/0.42
			VH	3, 4, 3			
		Nppa	DH	4, 4, 3	-4.34/-6.03/-0.938	6/6/5	0.0049/0.0009/0.39
			VH	4, 4, 4			
		Oprl1	DH	3, 3, 4	-1.78/-2.78/-0.649	4/4/6	0.15/0.050/0.54
			VH	3, 3, 4			
		Penk	DH	3, 4, 3	-1.40/-0.884/-0.455	10/12/10 (in duplicate except VH-P14)	0.19/0.39/0.66
			VH	4, 3, 3			
		Rxfp3	DH	4, 4, 4	-2.24/-3.15/-3.80	6/6/6	0.066/0.020/0.0090
			VH	4, 4, 4			
		Sstr1	DH	4, 4, 3	-3.12/-3.80/-0.351	6/6/4	0.021/0.0089/0.74
			VH	4, 4, 3			
	behavior fear response	Htr2c	DH	4, 4, 3	-1.52/-2.15/-1.68	6/7/4	0.18/0.068/0.17
			VH	4, 5, 3			
		Lypd1	DH	3, 3, 3	-0.911/-1.10/-0.801	5/5/5	0.40/0.32/0.46
			VH	4, 4, 4			
		Npy2r	DH	3, 4, 3	-4.43/-5.10/-3.59	5/6/4	0.0068/0.0022/0.023
			VH	4, 4, 3			
		Penk	DH	4, 3, 4	-1.27/-1.02/-0.694	13/11/12 (in duplicate except DH-P14, P45 & VH-P28)	0.23/0.33/0.50
			VH	4, 4, 4			
